# Factors influencing adolescents’ healthy pregnancy preparation behavior: a cross-sectional gender comparison applying the health belief model

**DOI:** 10.1186/s12978-022-01392-z

**Published:** 2022-04-05

**Authors:** Hae Won Kim, Saem Yi Kang, Jieun Kim

**Affiliations:** 1 Center for Human-Caring Nurse Leaders for the Future by Brain Korea 21 (BK 21) Four Project, College of Nursing, Research Institute of Nursing Science, Seoul National University, Seoul, 03080 Republic of Korea; 2 College of Nursing, Gyeongsang National University, Jinju, 52727 Republic of Korea; 3 Red Cross College of Nursing, Chung-Ang University, 84 Heukseok-ro, Dongjak-gu, Seoul, 06974 Republic of Korea

**Keywords:** Adolescent, Health promotion/wellness behaviors, Health beliefs, Lifestyle change, Reproductive health

## Abstract

**Background:**

Interventions to prepare for a healthy pregnancy from an early age can ensure the health of both mother and child. This study aims to compare the factors associated with healthy pregnancy preparation behavior (HPPB) among male and female adolescents.

**Methods:**

A total of 690 Korean adolescents participated in this cross-sectional study from July 11 to August 24, 2018. Determinants of the likelihood of engaging in HPPB were described using hierarchical regression about the importance of and confidence in HPPB, the gender equality related to pregnancy and birth, and the health belief model (HBM) constructs about HPPB.

**Results:**

Smoking experience (β = − 0.18, *p* < 0.001 for boys, β = − 0.25, *p* < 0.001 for girls), and HBM constructs were identified as factors correlated with HPPB in both genders. The significant factors in boys were perceived susceptibility (β = − 0.13, *p* = 0.005), perceived severity (β = 0.12, *p* = 0.015), perceived benefits (β = 0.23, *p* < 0.001), and perceived barriers (β = − 0.18, *p* < 0.001), whereas the corresponding factors in girls were perceived severity (β = 0.20, *p* = 0.001), and perceived barriers (β = − 0.23, *p* < 0.001). The importance of HPPB was identified as a factor only among girls (β = 0.19, *p* = 0.005), while confidence in HPPB (β = 0.12, *p* = 0.401), gender equality related to pregnancy and childbirth (β = − 0.20, *p* = 0.001 for women’s responsibility variable, β = 0.14, *p* = 0.018 for men’s responsibility variable), and alcohol (β = − 0.10, *p* = 0.022) were factors identified only among boys.

**Conclusions:**

The gender differences in opinions on HPPB identified in this study can help nurses and community health care professionals recognize issues for which they can develop and implement preventive interventions. For healthy pregnancy preparation, interventions based on HBM constructs and smoking should be presented for both male and female adolescents. Imparting education to females on the importance of HPPB and to males on confidence in HPPB, gender equality related to pregnancy and childbirth, and alcohol consumption, should be emphasized. In addition, as perceived susceptibility may be low in a disease prevention model using the health belief model, it is necessary to prioritize increasing the perceived susceptibility of school-age children as an intervention.

## Plain Language Summary

In the context of pregnancy preparation, attention has recently shifted from the period just before pregnancy to the early years, or even throughout one’s life. The World Health Organization identifies adolescents as requiring specific attention. Adolescents’ health behaviors can impact their adulthood years and have significant repercussions on the health of the future generation. In addition, recent studies consistently report that the preparedness of both men and women is essential to pregnancy preparation. Therefore, it is now time to present a new strategy by identifying and grasping the different factors that influence the two genders. This study can raise awareness of healthy pregnancy preparation behavior from adolescence onwards of both genders. There were differences in factors affecting HPPB by gender. For healthy pregnancy preparation, interventions based on HBM constructs and smoking should be presented for both male and female adolescents. Imparting education to females on the importance of HPPB (β = 0.19, *p* = 0.005) and males on confidence in HPPB (β = 0.12, *p* = 0.401), gender equality related to pregnancy and childbirth (β = − 0.20, *p* = 0.001 for women’s responsibility variable, β = 0.14, *p* = 0.018 for men’s responsibility variable), and alcohol consumption (β = − 0.10, *p* = 0.022) should be emphasized; and effective healthy pregnancy preventive strategies should be developed based on these findings. Interventions to prepare for a healthy pregnancy from an early age can ensure the health of both mother and child.

## Background

In the context of pregnancy preparation, attention has recently shifted from the period just before pregnancy to the early years, or even throughout one’s life [1, 2]. The International Federation of Gynecology and Obstetrics recommends that maternal health be part of a life course approach to dealing with women’s overall health [3]. The CDC (2020a) stated that planning for pregnancy relates to people becoming and staying healthy overall, throughout their lives, and presented 10 actions to achieve that state. However, studies show that many young people do not plan or prepare for pregnancy [4], with only a small proportion planning a pregnancy following lifestyle recommendations such as intake of folic acid and cessation of smoking and alcohol consumption [5, 6]. This causes the health of the fetus, mother, and, eventually, the family to deteriorate [7, 8].

The WHO identifies adolescents as requiring specific attention . Adolescents’ health behaviors can impact their adulthood years and have significant repercussions on the health of the future generation [3]. One follow-up study found that adolescents who were physically active had more positive outcomes in terms of their children’s birth weight [9]. More than eight out of 10 adults who regularly smoke report having started smoking in adolescence [10]. Increasing evidence reveals that adolescence is a crucial time for establishing health behaviors that affect the conception period; thus, there is a need to identify specific risk and protective factors.

Recent studies consistently report that the preparedness of both men and women is essential to pregnancy preparation. The CDC (2020a) defines preconception health as the health of women and men during their reproductive years or the years during which they can have a child. Another definition states that both genders should be prepared in order to ensure a healthy pregnancy [11]. However, studies on the risk factors for reproductive planning have focused mainly on women [12]. Therefore, it is now time to present a new strategy by identifying and grasping the different factors that influence the two genders. The health belief model (HBM) has been used to predict the likelihood of individuals engaging in health behaviors [13]. It assumes that the participation of individuals in the prevention, early detection, and treatment of a specific health problem depends on their perception [13]. As it has conventionally been chosen to study health promotion, it is also applicable within the context of reproductive health [14, 15]. The health beliefs and motivations of young people also directly relate to the utilization of available healthcare services such as preconception care [14].

Although awareness of the need for early intervention and gender differences related to healthy pregnancy preparation is increasing, no research has been conducted on these topics. Most other related studies have focused only on women [12, 16], with some studies examining unintended pregnancy, human immunodeficiency virus infection, and chronic diseases among adolescents [17]. It is imperative to lay the groundwork for and raise awareness about the preparation for a healthy pregnancy. Therefore, in this study, we aim to compare the factors associated with healthy pregnancy preparation behavior (HPPB) among male and female adolescents by applying the HBM.

## Methods

### Design and sampling

This cross-sectional study was conducted among male and female Korean adolescents. Convenience sampling was used to recruit participants from two high schools in Gyeonggi-do and Gyeongsangbuk-do provinces. Data analysis was performed on 690 participants, excluding 29 who did not respond, 29 who gave incomplete answers, and six who did not give consent. Our sample size was found to be sufficient based on a power analysis calculated by G*Power using linear multiple regression with an effect size of 0.15, power of 0.95, and 18 predictors considering a 20% dropout rate for the two gender groups.

### Measurements

#### HBM constructs about HPPB

HBM constructs about HPPB were assessed using a questionnaire developed by the researchers based on the pregnancy planning actions suggested by the CDC [18] and family planning objectives for adolescents based on Healthy People 2030 [19]. They include (a) alcohol abstinence, (b) smoking cessation, (c) contraceptive practice, and (d) sexually transmitted infection (STI) prevention and are characterized by changeable lifestyle behaviors. These items were also related to perceived susceptibility (reflecting the individual’s thoughts about the possibility of poor fetal and maternal health), perceived severity (reflecting the individual’s thoughts on how serious and problematic poor fetal and maternal health are), perceived benefits (reflecting the individual’s thoughts about how HPPB can improve the health of fetus and mother), perceived barriers (reflecting the individual’s concerns about the cost of engaging in that health behavior), and the likelihood of engaging in HPPB (reflecting the individual’s thoughts about whether one has the will to prepare for a healthy pregnancy). Items were rated on a four-point Likert scale (1 = *not at all*, 2 = *slightly*, 3 = *very*, 4 = *extremely*). The internal consistency of items for this questionnaire was estimated using Cronbach’s alpha, and value of 0.70–0.90 were considered acceptable (Cronbach’s alpha = 0.75 for perceived susceptibility, 0.78 for perceived severity, 0.87 for perceived benefits, 0.61 for perceived barriers, 0.72 for likelihood of engaging in HPPB). A panel of experts (a nursing professor, nursing experts, and PhD students) confirmed the instrument’s content validity.

#### Importance of and confidence in HPPB

The importance of and confidence in HPPB were assessed based on the CDC’s (2020b) recommendations for pregnancy planning [18] and family planning objectives for adolescents based on Healthy People 2030 [19]. Items included (a) proper contraceptive practice, (b) planning in advance for pregnancy and childbirth, (c) abstinence until becoming an adult, (d) not engaging in binge drinking (e) not smoking (f) preventing and managing STIs, (g) maintaining proper weight and physical activity, (h) watching out for harmful chemicals or environment, (i) asking for help if faced with abusive language or physical or sexual violence, (j) maintaining mental health, and (k) getting a vaccine necessary for health maintenance. These 11 items were rated from 0 to 100, with higher mean scores indicating greater importance of and confidence in HPPB. This questionnaire demonstrated good internal reliability and validity (Cronbach’s alpha = 0.85 for importance and 0.87 for confidence). The same panel of experts confirmed its content validity.

#### Gender equality related to pregnancy and birth

Gender equality related to pregnancy and birth was assessed through a questionnaire developed based on a concept analysis about gender egalitarianism for adolescents [20]. According to the concept analysis, gender equality was defined as an attitude that does not include stereotypes about the abilities, attributes, and roles of men and women in relation to pregnancy and childbirth, and recognizes the need for institutions and policies to correct gender discrimination in society. Participants were asked about the extent to which women and men are responsible for pregnancy and childbirth, childrearing, efforts for a healthy pregnancy, occurrence of unwanted pregnancy, complications during pregnancy, and high-risk pregnancy. The 10 items were rated on a five-point Likert scale (1 = *not at all*, 2 = *slightly*, 3 = *moderately*, 4 = *very*, 5 = *extremely*). This questionnaire demonstrated good internal reliability and validity (Cronbach’s alpha = 0.83). The same panel of experts confirmed the content validity.

#### General characteristics

Demographic characteristics comprised age, religion, living status, and economic status. Health behavior characteristics comprised alcohol consumption experience, smoking experience, and human papillomavirus vaccine (HPV) status based on a checklist of pregnancy planning for adolescents. In addition, sex-related characteristics comprised sex education experience and sexual experience.

### Ethical considerations

Ethical approval was obtained from the Institutional Review Board of Seoul National University (IRB No. 1806/003-006). Permission to conduct the study was obtained from the principals of the schools. The assistant researcher explained the study purpose and method to the students. If they agreed to participate, they signed written consent and legal representation forms. The participants were assured about the anonymity of their data, their privacy was guaranteed, and they were informed that they could withdraw their participation at any time. Individuals who were parents, did not agree to sign the forms, or did not want to participate were excluded. As the questionnaires contained sensitive information such as sexual experience, they were submitted in an unidentified document bag to ensure security of the content.

### Data collection and analysis

Data were collected from July 11 to August 24, 2018. The adolescents received a small gift as a reward for participation. All statistical analyses were performed using SPSS version 23 (IBM Corp, Armonk, NY, USA). General characteristics, importance of and confidence in HPPB, gender equality related to pregnancy and birth, and HBM constructs about HPPB were described using means and standard deviations (SDs) for numeric variables and frequency and percentage for categorical covariates. Gender differences in all variables were described using independent t-tests for numeric variables and chi-squared tests for categorical covariates. Correlates of the likelihood of adolescents engaging in HPPB were described using hierarchical regression. Statistical significance was set at *p* < 0.05.

## Results

### Participant characteristics

Participants’ characteristics are presented in Table 1. Among the 690 participants, 416 were boys and 274 were girls. Their mean age was 16.8 years (*SD* = 1.0); 36.4% followed a religion and 28.2% were satisfied with their academic achievement. Additionally, 92.9% were living with family, 93.2% had middle-to-high economic status, 43.2% had alcohol consumption experience, 19.9% had smoking experience, 7.8% had received the HPV vaccine, 80.7% had sexual education experience, and 91.7% did not have sexual experience. Boys were more likely than girls to have smoking experience (χ^2^ = 14.32, *p* < 0.001) and be satisfied with their academic achievement (χ^2^ = 20.45, *p* < 0.001). More girls than boys, however, received the HPV vaccine (χ^2^ = 15.42, *p* < 0.001).

**Table 1:** Participant characteristics (N = 690)

Variables	Total	Boys (n = 416)	Girls (n = 274)	95% CI		χ^2^ or t(*P*)
	n (%) or M ± SD			Lower	Upper	
Sociodemographic characteristics						
Age (years)	16.8 ± 1.0	16.9 ± 1.0	16.8 ± 0.9			0.73 (0.467)
15	66 (9.6)	46 (11.0)	20 (7.3)			
16	179 (25.9)	99 (23.8)	80 (29.2)			
17	245 (35.5)	138 (33.2)	107 (39.1)			
18	200 (29.0)	133 (32.0)	67 (24.4)			
Religion						0.07 (0.787)
No	439 (63.6)	263 (63.2)	176 (64.2)			
Yes	251 (36.4)	153 (36.8)	98 (35.8)			
Academic achievement						20.45 (< 0.001)
Dissatisfied	166 (24.1)	78 (18.8)	88 (32.1)			
Neither dissatisfied nor satisfied	329 (47.7)	201 (48.3)	128 (46.7)			
Satisfied	195 (28.2)	137 (32.9)	58 (21.2)			
Living condition						0.03 (0.870)
Without family	49 (7.1)	29 (7.0)	20 (7.3)			
With family	641 (92.9)	387 (93.0)	254 (92.7)			
Economic status						2.28 (0.320)
Low	47 (6.8)	33 (7.9)	14 (5.1)			
Middle	515 (74.6)	309 (74.3)	206 (75.2)			
High	128 (18.6)	74 (17.8)	54 (19.7)			
Health behavior characteristics						
Alcohol consumption experience						2.15 (0.143)
No	392 (56.8)	227 (54.6)	165 (60.2)			
Yes	298 (43.2)	189 (45.4)	109 (39.8)			
Smoking experience						14.32 (< 0.001)
No	553 (80.1)	314 (75.5)	239 (87.2)			
Yes	137 (19.9)	102 (24.5)	35 (12.8)			
HPV vaccine						15.42 (< 0.001)
No	636 (92.2)	397 (95.4)	239 (87.2)			
Yes	54 (7.8)	19 (4.6)	35 (12.8)			
Sex-related behavior characteristics						
Sexual education experience						0.39 (0.530)
No	133 (19.3)	77 (18.5)	56 (20.4)			
Yes	557 (80.7)	339 (81.5)	218 (79.6)			
Sexual experience						0.15 (0.700)
No	633 (91.7)	383 (92.1)	250 (91.2)			
Yes	57 (8.3)	33 (7.9)	24 (8.8)			
Importance of and confidence in healthy pregnancy preparation behavior						
Importance (range 0–100)	92.25 ± 10.09	92.02 ± 10.55	92.60 ± 9.36	− 2.12	0.96	− 0.74 (0.459)
Confidence (range 0–100)	87.98 ± 13.39	88.22 ± 13.31	87.61 ± 13.53	− 1.44	2.66	0.58 (0.559)
Gender equality related to pregnancy and birth						
Women’s responsibility	12.51 ± 3.75	13.12 ± 3.58	11.59 ± 3.82	0.97	2.09	5.35 (< 0.001)
Men’s responsibility	15.33 ± 3.63	15.31 ± 3.27	15.37 ± 4.13	− 0.61	0.50	− 0.20 (0.843)
HBM constructs about healthy pregnancy preparation behavior						
Perceived susceptibility	7.23 ± 2.62	7.37 ± 2.83	7.09 ± 2.27	− 0.12	0.68	1.37 (0.172)
Perceived severity	13.71 ± 1.93	13.60 ± 2.03	13.87 ± 1.76	− 0.56	0.02	− 1.80 (0.072)
Perceived benefits	13.67 ± 1.90	13.76 ± 1.93	13.53 ± 1.84	− 0.05	0.53	1.61 (0.109)
Perceived barriers	7.30 ± 2.14	7.27 ± 2.33	7.35 ± 1.82	− 0.41	0.24	− 0.52 (0.602)
Likelihood of healthy pregnancy preparation behavior	13.54 ± 2.06	13.56 ± 2.03	13.53 ± 2.11	− 0.28	0.35	0.22 (0.824)

*M* mean; *SD* standard deviation; *CI* confidence interval; *HPV* human papillomavirus; *HBM* health belief model

Participants’ mean scores on the importance of and confidence in HPPB were 92.25 (*SD* = 10.09) and 87.98 (*SD* = 13.39) out of 100, respectively. The responsibility scores of girls and boys were 12.51 (*SD* = 3.75) and 15.33 (*SD* = 3.63), respectively, with more boys than girls perceiving that women have more responsibility related to pregnancy and childbirth (*t* = 5.35, *p* < 0.001). There were no gender differences in the HBM constructs about HPPB.

### Correlates of the likelihood of engaging in HPPB

Factors associated with the likelihood of adolescents engaging in HPPB are presented in Table 2. Sociodemographic, health behavior, and sex-related behavior characteristics were entered in step 1. Smoking experience was significantly associated with the likelihood of engaging in HPPB (β = − 0.27, *p* < 0.001 for boys, β = − 0.26, *p* < 0.001 for girls). The importance of and confidence in HPPB were entered in step 2; the significant factors in boys were smoking experience (β = − 0.22, *p* < 0.001), importance of HPPB (β = 0.14, *p* = 0.031), and confidence in HPPB (β = 0.21, *p* = 0.001), whereas the corresponding factors in girls were smoking experience (β = − 0.25, *p* < 0.001) and the importance of HPPB (β = 0.22, *p* = 0.003). Gender equality related to pregnancy and birth were entered in step 3; the significant factors in boys were smoking experience (β = − 0.21, *p* < 0.001), confidence in HPPB (β = 0.22, *p* = 0.001), women’s responsibility (β = − 0.20, *p* = 0.001), and men’s responsibility (β = 0.14, *p* = 0.018), whereas the corresponding factors in girls were smoking experience (β = − 0.25, *p* < 0.001) and importance of HPPB (β = 0.22, *p* = 0.003). In step 4, HBM constructs were included and the final model was significant (F = 14.41, *p* < 0.001 for boys, F = 6.26, *p* < 0.001 for girls) with a significantly better predictor than the model in steps 1, 2, and 3 (Adjusted R^2^ = 0.37 for boys, Adjusted R^2^ = 0.26 for girls). The significant factors in boys were alcohol consumption experience (β = − 0.10, *p* = 0.022), smoking experience (β = − 0.18, *p* < 0.001), confidence in HPPB (β = 0.12, *p* = 0.401), perceived susceptibility (β = − 0.13, *p* = 0.005), perceived severity (β = 0.12, *p* = 0.015), perceived benefits (β = 0.23, *p* < 0.001), and perceived barriers (β = − 0.18, *p* < 0.001), whereas the corresponding factors in girls were smoking experience (β = − 0.25, *p* < 0.001), importance of HPPB (β = 0.19, *p* = 0.005), perceived severity (β = 0.20, *p* = 0.001), and perceived barriers (β = − 0.23, *p* < 0.001).

**Table 2:** Correlates of the likelihood of engaging in healthy pregnancy preparation behavior

Variables	Boys (n = 416)							
	Model 1		Model 2		Model 3		Model 4	
	B	β	B	β	B	β	B	β
(Constant)	13.78		8.61		9.52		8.20	
Age (years)	− 0.01	− 0.00	− 0.01	− 0.01	− 0.03	− 0.02	− 0.03	− 0.39
Religion	0.11	0.03	0.09	0.02	0.06	0.01	0.06	0.38
Academic achievement	0.08	0.03	− 0.01	− 0.00	0.02	0.01	0.03	0.28
Living condition	− 0.53	− 0.07	− 0.50	− 0.06	− 0.51	− 0.06	− 0.46	− 1.44
Economic status	0.19	0.05	0.15	0.04	0.15	0.04	0.13	0.83
Alcohol consumption experience	− 0.33	− 0.08	− 0.22	− 0.05	− 0.26	− 0.06	− 0.42	− 2.30*
Smoking experience	− 1.29	− 0.27***	− 1.06	− 0.22***	− 1.01	− 0.21***	− 0.83	− 3.78***
HPV vaccine	− 0.09	− 0.01	− 0.06	− 0.01	− 0.03	− 0.00	0.04	0.10
Sexual education experience	0.33	0.06	0.30	0.06	0.34	0.07	0.11	0.51
Sexual experience	0.24	0.03	0.32	0.04	0.22	0.03	− 0.13	− 0.43
Importance of HPPB			0.03	0.14*	0.02	0.11	0.02	1.51
Confidence in HPPB			0.03	0.21**	0.03	0.22**	0.02	2.07*
Women’s responsibility					− 0.55	− 0.20**	− 0.27	− 1.80
Men’s responsibility					0.44	0.14*	0.23	1.41
Perceived susceptibility							− 0.09	− 2.85**
Perceived severity							0.12	2.44*
Perceived benefits							0.24	4.66***
Perceived barriers							− 0.16	− 3.88***
R^2^ (Δ R^2^)	0.11		0.21(0.10)		0.23(0.02)		0.40(0.17)	
*Adj* R^2^	0.09		0.19		0.20		0.37	
F(*P*)	4.84***		8.83***		8.54***		14.41***	

Variables	Girls (n = 274)							
	Model 1		Model 2		Model 3		Model 4	
	B	β	B	β	B	β	B	β
(Constant)	14.94		11.15		11.36		10.52	
Age (years)	− 0.19	− 0.08	− 0.24	− 0.10	− 0.26	− 0.11	− 0.27	− 0.12
Religion	0.12	0.03	0.02	0.00	0.02	0.00	− 0.15	− 0.04
Academic achievement	0.28	0.10	0.20	0.07	0.22	0.07	0.20	0.07
Living condition	0.53	0.07	0.37	0.05	0.36	0.05	0.31	0.04
Economic status	0.26	0.06	0.24	0.05	0.22	0.05	0.31	0.07
Alcohol consumption experience	− 0.21	− 0.05	− 0.19	− 0.04	− 0.18	− 0.04	− 0.32	− 0.07
Smoking experience	− 1.66	− 0.26***	− 1.58	− 0.25***	− 1.56	− 0.25***	− 1.57	− 0.25***
HPV vaccine	0.27	0.04	0.27	0.04	0.25	0.04	0.22	0.04
Sexual education experience	0.55	0.11	0.55	0.11	0.56	0.11	0.37	0.07
Sexual experience	0.25	0.03	0.49	0.07	0.47	0.06	0.25	0.03
Importance of HPPB			0.05	0.22**	0.05	0.22**	0.04	0.19**
Confidence in HPPB			0.00	0.02	0.00	0.02	0.00	− 0.00
Women’s role and/or responsibility					− 0.21	− 0.08	− 0.16	− 0.06
Men’s role and/or responsibility					0.24	0.09	0.13	0.05
Perceived susceptibility							0.02	0.02
Perceived severity							0.24	0.20**
Perceived benefits							0.06	0.05
Perceived barriers							− 0.27	− 0.23***
R^2^ (Δ R^2^)	0.13		0.18 (0.05)		0.19 (0.01)		0.31 (0.12)	
*Adj* R^2^	0.10		0.15		0.14		0.26	
F(*P*)	4.05***		4.90***		4.27***		6.26***	

*HPV* human papillomavirus; *HBM* health belief model

*p < 0.05, **p < 0.01, ***p < 0.001

## Discussion

This study provides novel information by identifying the likelihood of adolescents engaging in HPPB in the future. Importantly, it reveals the common and differentiating factors between genders regarding the likelihood of engaging in HPPB. Consistent with previous research, we observed gender differences with regard to academic achievement, smoking experience, HPV vaccination, and women’s responsibility toward pregnancy and birth [21]. It is important to focus on these factors as they lead to differences in healthy pregnancy behaviors between men and women, such as safe sexual behaviors [21]. In particular, it should be noted that this study is the first to confirm that the perception of gender equality related to pregnancy and childbirth is still low for adolescents, a group that represents the new generation. Another recent study found that young men’s perceptions related to their preparation for pregnancy are still low [22]. Traditionally, HPPB is viewed as the domain and responsibility of women, leading to poor health among following generations [23]. The fact that gender continues to be a significant differentiating factor in studies related to attitudes about roles related to pregnancy and childbirth must be recognized by nurses and public policy developers.

Past studies have confirmed that the perceived importance of and confidence in behaviors are important factors impacting the strategy of health promotion activities [24]. In model 2 of the hierarchical regression analysis, the importance and reliability of HPPB were added in addition to the general characteristics. As expected, they significantly increased the likelihood of engaging in HPPB, although gender differences were identified: the likelihood of engaging in HPPB increased with higher perceived importance in female adolescents and higher confidence in male adolescents. In a previous study, confidence in men’s healthy pregnancy readiness was found to prevent teenage pregnancy [25]. It is important to emphasize the importance of high-risk pregnancies for women and encourage the development of confidence in HPPB for men as they are educated about HPPB. Although providing information regarding health behaviors by itself may be insufficient to ensure their implementation, it proposes a specific approach to preconception education.

There were differences in gender equality related to pregnancy and childbirth among adolescents, which revealed gender differences in their likelihood of engaging in HPPB. In model 3 in the hierarchical regression, gender equality related to pregnancy and childbirth was a significant factor only for male adolescents. Male adolescents who think women play a greater role in preparing for a healthy pregnancy have a reduced likelihood of engaging in HPPB. For female adolescents, this difference in perception was not associated with their likelihood of engaging in HPPB. In model 4 in the hierarchical regression with the added HBM structure, gender equality related to pregnancy and childbirth was not a significant variable, which requires careful interpretation. However, as most research has studied healthy pregnancy readiness with a focus on women [16], the present results provide new and encouraging evidence. Previous studies have conducted descriptive surveys of the current status of awareness of preconception care according to gender [26], but this study confirmed the relationship with the possibility of actual behavioral change. Studies on conception care have reported that the majority of men believe they share equal decision-making responsibility with their female partners [27]; however, parallel measures do not exist for preconception care. If men continue to take preparing for a healthy pregnancy lightly, it can have adverse health outcomes for mothers and children [11]. Therefore, in order to encourage healthy pregnancy preparation among men, it is necessary to continuously inform them that the responsibility for these health behaviors lies with both women and men.

The hierarchical regression analysis showed that the explanatory power increased significantly when HBM constructs were added to the general characteristics, importance of and confidence in HPPB, and gender equality related to pregnancy and childbirth in model 4. This result aligns with the findings of another study, which revealed that HBM constructs can explain the stage of nutrition behavioral changes for women receiving preconception care [12]. Most previous HBM studies on conception care have been conducted on women during pregnancy, with no studies on men [12, 28]. Among HBM constructs, perceived susceptibility, perceived severity, perceived benefits, and perceived barriers were all significant factors for male adolescents, and perceived severity and perceived barriers were significant factors for female adolescents. It is of paramount importance to consider these gender differences when developing effective preventive nursing interventions for healthy pregnancy preparation. Since HBM and several kinds of healthy pregnancy behaviors have showed a strong correlation [28, 29], the results of this study could be a reliable basis for the newly identified relationship.

In Model 1 of the hierarchical regression analysis, among the health behavior characteristics, the possibility of participating in HPPB showed both commonalities and differences according to gender. The consumption of alcohol and tobacco in male adolescents and consumption of tobacco in female adolescents was associated with the likelihood of engaging in HPPB. Numerous previous studies have emphasized the life course approach to non-communicable disease, with some highlighting its importance in preparing for a healthy pregnancy [30]. In a previous longitudinal study, smoking before pregnancy led to smoking during pregnancy, which increased the risk of miscarriage and delayed fetal development [23]. Additionally, smoking before pregnancy tends to persist after pregnancy, causing secondhand smoke inhalation in infants. There have also been follow-up studies of women’s alcohol consumption from pre-pregnancy to pregnancy [31]. Although there have been no previous studies on men, there is sufficient proof of how men’s unhealthy habits affect their own and their children’s health outcomes from a life course perspective [32]. It is important to extend the present findings regarding male adolescents to determine whether and how alcohol intake during adolescence actually affects the health of spouses during pregnancy as well as the health of offspring. Abstaining from alcohol and tobacco is an essential HPPB [33]. Therefore, preventing these experiences early will have a positive effect.

Other participant characteristics such as HPV vaccination and sexual experience were not associated with the likelihood of engaging in HPPB in both boys and girls [21]. In previous studies, contraception rather than sexual experience showed differences in preventive behavior for STIs and abortion [34], showing the similarity to this study’s results. In another cross-sectional study, a significant relationship was found between HPV vaccination and HPPB like smoking, which is inconsistent with our findings. Health behavioral characteristics such as HPV vaccination and sexual experience, which were not significant in this study, were considered to have no statistical difference as they were indicators with a small “yes” distribution. This should be reanalyzed in studies with large sample sizes. The main difference between this study and previous research is that the present participants were adolescents who were not planning a pregnancy in the near future. Exploring adolescents’ various characteristics and behaviors can lead to preparing the HPPB.

## Limitations and implications

This study utilized a cross-sectional design to examine the relationships between the likelihood of adolescents engaging in HPPB and its influencing factors. Although various participant characteristics were taken into consideration, it is difficult to generalize the findings because the participants were selected through convenience sampling in only one city, not on a national scale. In consideration of the limitations mentioned previously, we propose that, in the future, a large number of individuals from across the country with varying demographic characteristics should be considered, utilizing other sampling techniques. Longitudinal designs are required to properly demonstrate causality. Additionally, future studies should replicate this study to determine whether this likelihood and the influencing factors identified result in actual HPPB. It is necessary to collect evidence regarding entry into healthy pregnancies by confirming these findings through studies on diverse health promotion topics and age groups, including adolescents.

Although providing information about health behaviors alone may not be sufficient to facilitate their implementation, the current findings highlight the importance of awareness about the mechanism by which behaviors can be modified. In addition, this study presents feasible and applicable results. This study is one of the first to confirm the recently established concept of preconception as a period for early interventions. In addition, it investigates male adolescents’ perceptions of preparing for a healthy pregnancy, unlike traditional female-oriented research. These findings provide evidence for nurses and community health care professionals regarding the need for preventive interventions and directions to develop such interventions and improve the likelihood of adolescents engaging in HPPB.

## Conclusions

The present study details one of the first attempts to raise awareness by identifying the causes of HPPB in adolescence. Although the CDC and WHO have long emphasized this need, the lack of evidence of the importance of early intervention for HPPB has resulted in insignificant efforts to solve this challenge; thus, it had been difficult to be incorporated into health policy. The results of this study revealed that there were gender differences in the factors affecting HPPB, and effective healthy pregnancy preventive strategies should be developed based on these results. For healthy pregnancy preparation, interventions related to the HBM constructs and smoking should be offered for both male and female adolescents. Education on the importance of HPPB for girls and on confidence in HPPB, gender equality related to pregnancy and childbirth, and alcohol consumption for boys should be emphasized. A thorough understanding of HPPB has several implications for practices and policies for intergenerational health. The results of this study can help nurses and community health care professionals recognize the differences of opinion on HPPB between male and female adolescents and develop or provide appropriate interventions for HPPB.

## Data Availability

Due to the nature of this research, participants of this study did not agree for their data to be shared publicly, so supporting data is not available.

## Abbreviations

FIGO: Federation of Gynecology and Obstetrics
HBM: Health belief model
HPPB: Healthy pregnancy preparation behavior
STI: Sexually transmitted infection
